# Anodically Bonded Photoacoustic Transducer: An Approach towards Wafer-Level Optical Gas Sensors

**DOI:** 10.3390/s22020685

**Published:** 2022-01-17

**Authors:** Simon Gassner, Rainer Schaller, Matthias Eberl, Carsten von Koblinski, Simon Essing, Mohammadamir Ghaderi, Katrin Schmitt, Jürgen Wöllenstein

**Affiliations:** 1 Department of Microsystems Engineering (IMTEK), Albert-Ludwigs-Universität Freiburg, 79085 Freiburg im Breisgau, Germany; katrin.schmitt@imtek.uni-freiburg.de (K.S.); juergen.woellenstein@imtek.uni-freiburg.de (J.W.); 2 Infineon Technologies AG, 81549 Neubiberg, Germany; rainer.schaller@infineon.com (R.S.); matthias.eberl@infineon.com (M.E.); Amir.Ghaderi@infineon.com (M.G.); 3 Infineon Technologies Austria AG, 9500 Villach, Austria; Carsten.Koblinski@infineon.com; 4 Department of Electrical and Computer Engineering, Technical University of Munich, 80333 München, Germany; simon.essing@tum.de; 5 Fraunhofer IPM, 79110 Freiburg, Germany

**Keywords:** gas sensor, photoacoustic, pressure transducer, wafer-level, CO_2_

## Abstract

We present a concept for a wafer-level manufactured photoacoustic transducer, suitable to be used in consumer-grade gas sensors. The transducer consists of an anodically bonded two-layer stack of a blank silicon wafer and an 11 µm membrane, which was wet-etched from a borosilicate wafer. The membrane separates two cavities; one of which was hermetically sealed and filled with CO_2_ during the anodic bonding and acts as an infrared absorber. The second cavity was designed to be connected to a standard MEMS microphone on PCB-level forming an infrared-sensitive photoacoustic detector. CO_2_ sensors consisting of the detector and a MEMS infrared emitter were built up and characterized towards their sensitivity and noise levels at six different component distance ranging from 3.0 mm to 15.5 mm. The signal response for the sample with the longest absorption path ranged from a decrease of 8.3% at a CO_2_ concentration of 9400 ppm to a decrease of 0.8% at a concentration of 560 ppm. A standard deviation of the measured values of 18 ppm was determined when the sensor was exposed to 1000 ppm CO_2_.

## 1. Introduction

Over the last decade, the public interest in air pollution measurement has gradually increased, giving rise to high demand for air quality sensors [1,2]. There are various different approaches to classify the air quality based on the concentration measurement of air pollutants including either a specific or a mix of volatile organic compounds (VOC), total suspended particles (TSP), or relative humidity (RH) [3,4,5,6]. Moreover, the concentration of carbon dioxide (CO_2_) has been shown to be one of the most applicable indicators for indoor air quality. CO_2_ is a major indoor pollutant which, even at slightly elevated gas concentrations, leads to declining work performance and diminishing focus capacity in the human organism [7]. Monitoring CO_2_ levels indoors is, therefore, essential to achieve an optimal balance between maximizing human performance and minimizing the need for energy-expensive ventilation of indoor spaces. In automotive environments, CO_2_ can reach increased concentration levels with undesirable physiological effects in a very short period of time, due to exhaust gases entering the vehicle cabin or due to metabolically induced CO_2_ emissions by the passengers. Typical adverse effects are fatigue, drowsiness, and lethargy which can lead to a higher risk of traffic accidents [8].

Although the CO_2_ concentration limits inducing adverse effects in humans are not well defined, the standardly used value dates back to Max von Pettenkofer in 1858, who defined a hygienic limit of 1000 ppm CO_2_ [9]. The consensus is that monitoring and keeping the CO_2_ concentration as low as possible is beneficial for indoor air quality.

To date, various different gas sensing technologies are available to determine the CO_2_ content in the air, including, but not limited to, non-dispersive infrared (NDIR) spectrometry, photoacoustic spectroscopy (PAS), quartz crystal microbalance (QCM) sensors, and several technologies based on chemical interactions of gas with a sensing material [10,11,12,13,14,15]. Most sensors currently available on the market for consumer electronics are based on either NDIR or PAS, as they are the only mature techniques with high selectivity to CO_2_, which are still cheap and durable enough to be relevant. Both are based on the absorption of electromagnetic waves with a wavelength tailored to match the specific CO_2_ absorption wavelengths.

NDIR sensors directly measure gas quantities by detecting slight changes in the transmittance of infrared light in an absorption path between a source and a detector. For selectivity towards a target analyte, optical filters are used. However, these filters used in NDIR sensors can only be designed for optical bandwidths rather than for the absorption spectrum of a molecule. Thus, the sensor may not only be selective towards its target gas, but also towards interfering gases, such as humidity. Furthermore, these filters are prone to temperature variations as the optical properties of such filters change with temperature. The change of intensity is described by the Beer-Lambert-Law and is strongly dependent on the concentration of absorbing molecules in the path and the traveled distance of the light. NDIR sensors typically use pyroelectric or bolometer infrared detectors, which naturally have a relatively low signal-to-noise ratio (SNR). In order to get significant signal levels, these sensors use long absorption paths achieved by multiple reflections inside the housing and narrow optical filtering. Although there were successful optimizations of reflector geometries for the design of compact and still optically efficient NDIR sensors, these reflectors are rather costly to manufacture [16].

PAS sensors, on the other hand, make use of the photoacoustic effect, discovered by Bell and Roentgen in 1881, and sense CO_2_ indirectly by measuring sound [17,18]. These sensors detect gas concentrations by introducing periodically modulated light of a specific wavelength into a detection chamber in which a target gas absorbs precisely that wavelength. The molecules of the target gas get excited to higher energy levels by the absorption and generate thermal energy by colliding with molecules of a carrier gas. The thermal energy change in the closed chamber is translated into a pressure change, which is sensed by a microphone. The signal level of photoacoustic sensors correlates with the concentration of CO_2_ inside the sensor. However, as these sensors use microphones to detect the photoacoustic signal, optimized gas port geometries need to be used in order to prevent acoustical interference such as banging doors or loud conversations. These gas inlets cause an increase in the response time of PAS CO_2_ sensors [19,20].

Here, we investigate a new sensing concept which combines elements of both, NDIR and PAS sensors and includes a manufacturing approach for a detector module, which offers potential for miniaturized and very selective gas measurements. In contrast to NDIR detectors, the presented photoacoustic transducer is highly selective towards CO_2_ as only its CO_2_ filling absorbs infrared light. The detectors are simple to manufacture and therefore offer great potential for fast and low-cost production of miniaturized CO_2_ sensors. In addition, the approach also resolves the issue of acoustic interference by concept, as the microphone is embedded within a closed cavity, without increasing the response time of the sensor.

## 2. Materials and Methods

### 2.1. Sensor Concept

The core of the presented concept is the development of a photoacoustic transducer chip that allows the spatial separation of the acoustic sensor from the photoacoustic cell. The transducer consists of a gas-tight and gas-filled cavity, acting as an optical absorption layer that transfers the irradiated infrared light energy to acoustical pulses and a thin, flexible membrane that can pass these pulses to a second cavity containing a microphone.

**Figure 1:**
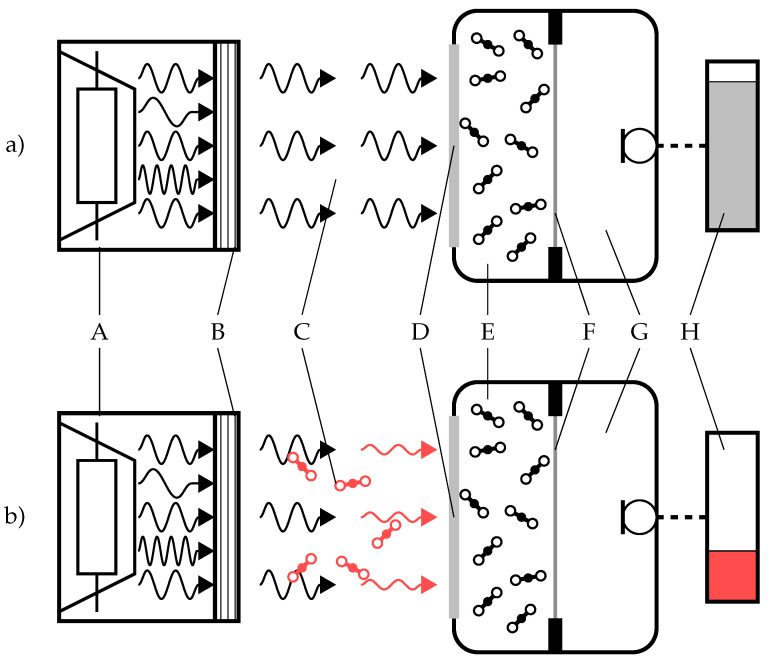
Conceptual sketch of the proposed sensing mechanism comprising: (A) pulsed IR-emitter (B) optical filter (optional) (C) optical absorption path (D) IR-transparent entrance window (E) hermetic cell with encapsulated CO_2_ (F) flexible membrane (G) acoustically tight cavity with microphone (H) sensor signal. (**a**) sensor without CO_2_ (**b**) sensor in presence of CO_2_.

Figure 1 illustrates a conceptual sketch of the sensing mechanism. A MEMS hotplate emits infrared (IR) pulses of defined frequency and pulse length, which may be filtered by means of an optical filter with a transmission spectrum matching the characteristic absorption band of CO_2_ at λ = 4.26 μm. Although this filter is not necessarily needed, it can prevent wavelengths different from those of the CO_2_ absorption spectrum to get absorbed in Silicon or glass parts of the transducer and thus increasing the performance. The pulses of IR light pass an optical absorption path, where they get partly absorbed by potentially present CO_2_ molecules.

After the absorption path, the light pulses enter a hermetically encapsulated absorption cell through an optically transparent and rigid window. The absorption cell is filled with CO_2_, which acts as a gas-specific absorption medium and therefore absorbs the IR light pulses with the characteristic absorption wavelengths of CO_2_. The absorbed energy gets transformed into acoustic waves by means of the photoacoustic effect. The photoacoustic effect itself consists of three different steps: The radiative absorption by the gas molecules, the successive relaxation of the molecules while increasing the total thermal energy in the gas, and—by means of the ideal gas law in a closed system—the resulting emergence of a pressure signal [20]. As the IR light pulses are modulated with a defined frequency, the pressure signal also follows this frequency. The more energy is absorbed in the absorption path, the lower the resulting intensity of the acoustic waves generated in the cell is.

One wall of the absorption cell consists of a thin and flexible, but gas-tight membrane, which slightly deforms with the pressure fluctuations and therefore allows the generated pressure pulses to be transmitted to the other side while maintaining the CO_2_ atmosphere inside the absorption cell. This way, the acoustic pulses can enter the second cavity, which needs to be only acoustically-tight, but not necessarily gas-tight. Inside this second cell, a MEMS microphone is placed, which detects the arriving acoustic pulses. The amplitude of the acoustic pulses is indirectly proportional to the number of CO_2_ molecules present in the absorption path.

The concept of using a slightly deforming membrane or diaphragm for gas sensing was first described by Golay as part of an infrared active pneumatic detector [21]. In contrast to the concept presented here, the membrane was utilized as a deforming mirror in an optical modulation circuit rather than as an acoustic transducer.

### 2.2. Manufacturing

**Figure 2:**
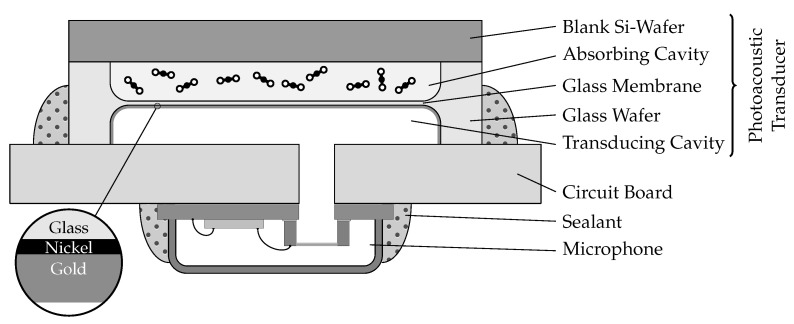
Proposed detector element comprising the photoacoustic transducer, a microphone, sealant compounds and a carrier PCB (not to scale).

Figure 2 depicts a schematic cross-section of the photoacoustic transducer. Its centerpiece is a thin, homogeneous glass membrane that has been wet-etched from a borosilicate glass wafer with a thickness of approximately 500 μm. A silicon nitride (SiN) hard mask was applied from the top and the bottom side to the glass wafer and was structured by lithography. At the openings of the hard mask, an anisotropic etch process symmetrically created a cavity on both sides of the wafer, leaving residual thin membranes with a diameter of approximately 3.6 mm. Defined by the duration of the etching, thicknesses between about 10 μm and 70 μm could be achieved. Within each membrane, the thickness tolerance was found to be ±1 μm. For investigation of the concept, we focused on samples with the thinnest membrane thickness, as they are the most flexible and have the highest mechanical compliance.

After etching, the glass wafer was anodically bonded to a blank silicon wafer, which was dry-polished in order to achieve the highest bond quality. Furthermore, for the purpose of maximizing the infrared light transmission, the used Si wafer was low doped and backside-grounded to a thickness of 250 μm. The anodic bond was formed at 300 °C while applying a CO_2_ bias atmosphere of 2 bar. Thus, after cooling down to ambient temperature, a CO_2_ filling of about 1 bar was captured in the gas-tight cavity between the glass membrane and silicon lid. After bonding, the wafer was mechanically sawed to form transducers of the size 5.5 mm by 5.5 mm.

**Figure A1:**
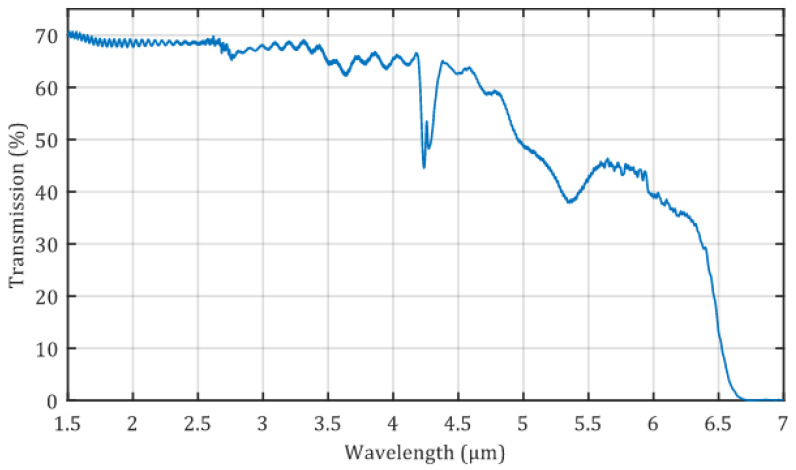
Background-compensated infrared transmission spectrum of the photoacoustic transducer. A characteristic dip at 4.26 μm indicates the presence of a high concentration of CO_2_ inside the cavity of the device.

Using Fourier transformation infrared (FTIR) spectroscopy, an absorbing behavior at the CO_2_ absorption bands was confirmed as shown in the transmission spectrum (Figure A1).The characteristic shape of the CO_2_ absorption at 4.26 μm was clearly visible with a relative absorption of about 15% to 20% compared to the background. The spectrum was limited at the lower end by the natural transparency of silicon and the capabilities of the used FTIR spectrometer and on the upper end by the borosilicate glass, which is nontransparent for light with wavelengths above 6.6 μm.

The singularized transducers were then treated by means of physical evaporation and deposition to form a nickel-gold coating as a nontransparent, reflecting layer for the infrared light. In this way, it was ensured that no IR light could hit and potentially disturb the microphone membrane and, additionally, that reflection caused a second pass of the light through the absorption cavity, increasing the absorption inside.

### 2.3. Experimental Setup

Figure 2 also depicts a sketch of the whole photoacoustic detector unit used in this research. Below the photoacoustic transducer, which was described in Section 2.2, a printed circuit board (PCB) carrying a MEMS-microphone is shown. The used analog microphone (IM73A135, Infineon Technologies AG, Munich, Germany) was soldered on the backside of a PCB for electrical connection. The photoacoustic transducer was mounted on the top side of the PCB, whilst ensuring that its transducing cavity was connected to the microphone membrane via a small channel in the PCB.

As an infrared light source of the emitter unit, a small MEMS hotplate embedded in a 4 × 4 × 2.25 mm^3^ package was used. The top wall of the package consisted of an infrared filter, which was tuned to match the dominant CO_2_ absorption band at its center wavelength of 4.26 μm. The IR emitter was soldered onto a second PCB which was mounted on the sensor evaluation system so that its surface was directly facing the photoacoustic transducer on the detector unit.

**Figure 3:**
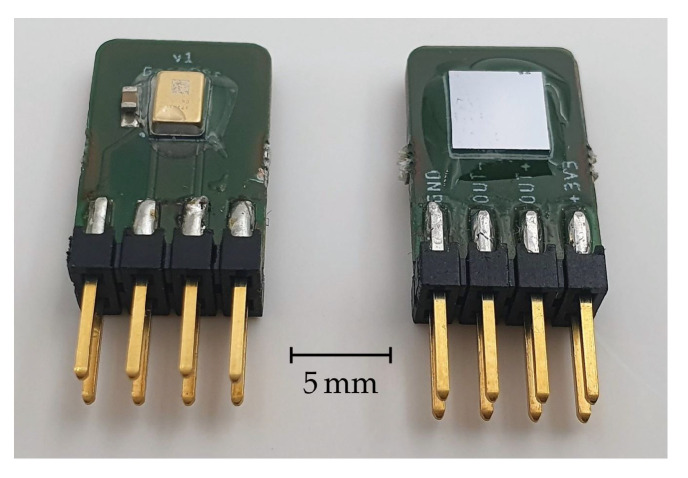
Detector module PCB with photoacoustic transducer (**right**, front side) and the MEMS microphone (**left**, back side).

Figure 3 shows a photograph of both sides of the detector PCB carrying a MEMS microphone, photoacoustic transducer, connectors, and the transparent epoxy glue, which was used to attach the photoacoustic transducer and to acoustically seal the gaps between components and PCB.

**Figure 4:**
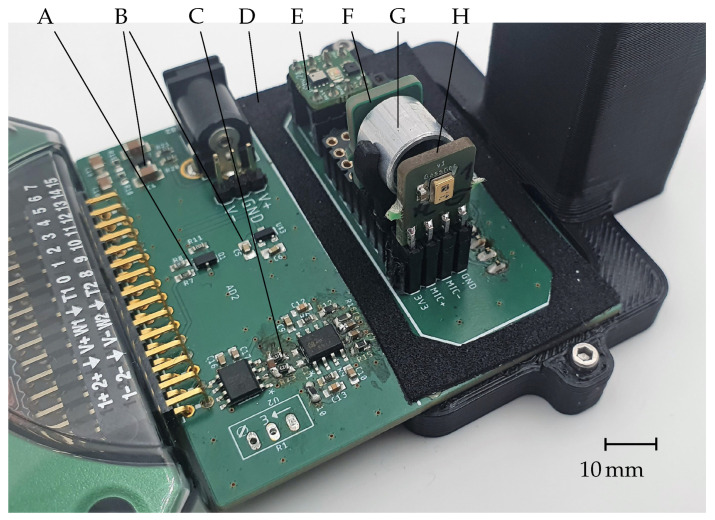
Sensor evaluation PCB with (A) MOSFET (B) power supply (C) amplifier stages (D) gasket (E) reference sensors (F) emitter module (G) reflector tube (H) detector module.

The distance between the upper surface of the emitter package and the detector could be set between 3.0 mm to 15.5 mm in intervals of 2.5 mm. To maximize the optical transmission from emitter to detector, aluminum tubes with matching lengths and an inner diameter of 8 mm were used as reflectors in between the sensor elements. The sensing area of the measurement system is surrounded by a gasket, which, together with a 3D-printed box with inner dimensions of 22 × 52 × 35 mm^3^, forms an enclosed box connected to a gas measurement system where different CO_2_ concentrations can be set. A photograph of the whole sensor assembly is shown in Figure 4.

The emitter was driven with a square signal by an n-channel metal-oxide-semiconductor field-effect transistor (MOSFET) with a frequency of 27 Hz for a duration of 1 s in every measurement period which lasted 5 s. The microphone output was a differential signal which was first pre-amplified and filtered by a second-order bandpass filter. It was configured to have a bandpass between 16 Hz and 210 Hz and an amplification gain of G = 9 (ADA4084-2, Analog Devices Inc., Wilmington, MA, USA). In a second amplification stage, the signal was amplified with an audio amplifier (SSM2019, Analog Devices Inc., Wilmington, MA, USA) with a gain of G = 370. The PCB was attached to a Digilent Analog Discovery 2 USB oscilloscope (Digilent, Pullman, WA, USA), which was used as a data acquisition system for controlling the sensor.

**Figure A2:**
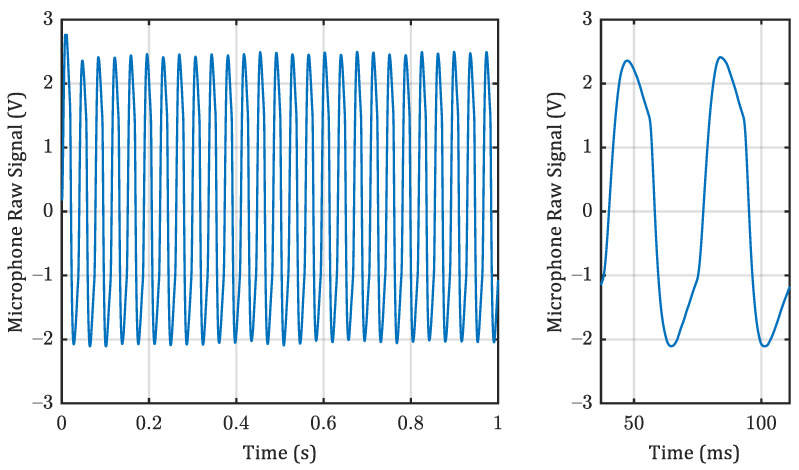
Raw microphone output signal over 1 s of sampling (**left**) and two periods (74 ms) of the signal (**right**).

The system acquired the microphone waveform differentially with a resolution of 14 bit at a sampling frequency of 10 kS/s. After the acquisition, the output waveform of the microphone and its amplification circuitry was processed in MATLAB (MATLAB version R2020b) by means of a discrete Fourier transformation in order to extract the specific spectral components from the discrete signal as described in [22]. For this calculation, a rectangular window with a period of 950 ms was applied. As the emitter excitation was carried out with a square signal, the microphone output resulted in a non-sinusoidal signal with multiple frequency components (raw signal in Appendix A, Figure A2). For this reason, the raw output value of the sensor system was defined as not only the amplitude of the spectral component at the excitation frequency of 27 Hz, but also the sum of its first and second harmonics. Adding the harmonics to the 27 Hz component was beneficial for the calculation of the sensor response and resulted in both, higher signal response and less signal noise. For further reduction of the sensor noise, a centered moving mean filter with a sliding window width of 12, equal to 1 min of sampling, was applied to the sensor output in postprocessing.

In addition to the actual sensor data acquisition, a secondary environmental data measurement system acquired information about relative humidity, temperature, pressure once in every measurement cycle. The CO_2_ concentration was measured with an additional, external industrial-grade NDIR sensor (M1440, COMET SYSTEM, s.r.o., Rožnov pod Radhoštěm, Czech Republic) in the exhaust flow of the gas testing bench, which served as a reference signal.

## 3. Results and Discussion

Using the setup described in the previous section, we measured and analyzed the sensor sample at six different absorption lengths (3.0 mm to 15.5 mm in intervals of 2.5 mm) with decreasing CO_2_ concentration steps ranging from about 9500 ppm down to 500 ppm. The measurement bench, which mixed CO_2_ and dry, synthetic air (80% N_2_ and 20% O_2_ ±2% ), allowed for a consistent gas flow of 850 cm^3^ min^−1^ through the sensor enclosure. Each concentration step was maintained for 10 min, followed by a flush step of 0 ppm, allowing to evaluate the sensor’s baseline after each concentration step. The temperature in the sensor enclosure during all measurements ranged from 31.8 °C to 34.3 °C with a mean temperature of 33.2 °C.

**Figure 5:**
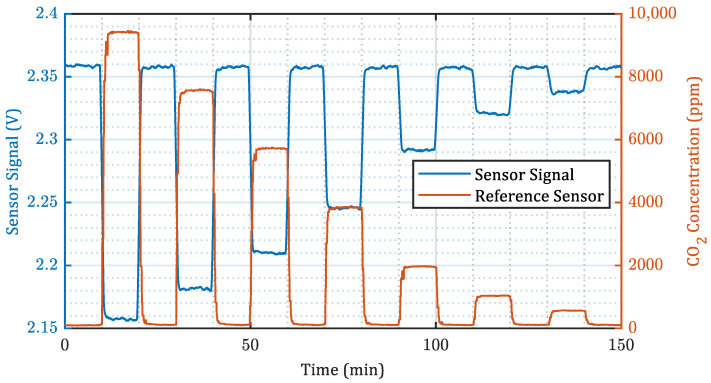
Measurement with an absorption distance of d = 15.5 mm showing 10 min steps with decreasing CO_2_ concentration together with the output of a NDIR reference sensor (12-times moving mean filter applied).

Figure 5 shows the absolute sensor signal with an absorption distance of 15.5 mm over a complete characterization procedure. Over the course of the measurement, the sensor signal repeatedly dropped in response to the application of the different CO_2_ concentration steps. The figure also shows the output of the reference sensor, for which a lower noise level compared to our sensor could be observed. However, in this regard, it has to be noted, that despite our sensor being an early prototype aiming for low-cost production of an integrated MEMS solution, it yields almost similar results compared to the well-established NDIR reference sensor, which is a larger, fully developed product.

The sensor prototype clearly responded with a lower signal to the increased number of CO_2_ molecules inside the absorption path, as a portion of the infrared light intensity already was absorbed there rather than inside the pressure transducer. The signal level at the lowest CO_2_ concentration applied ( 95 ppm) was found to be 2.37 V ± 0.70 mV. The signal response ranged from a decrease of about 197 mV (8.3%) at a concentration of 9400 ppm to a decrease of 18.8 mV (0.8%) at a concentration of 560 ppm. The sensitivity was calculated as signal response per decrease of 1000 ppm CO_2_ and thus ranged from 21.5 mV/1000 ppm for 9500 ppm to −41.7 mV/1000 ppm for 500 ppm. The first 2 min before and after each concentration change were ignored in the data processing in order to ensure a stabilized CO_2_ concentration.

**Figure 6:**
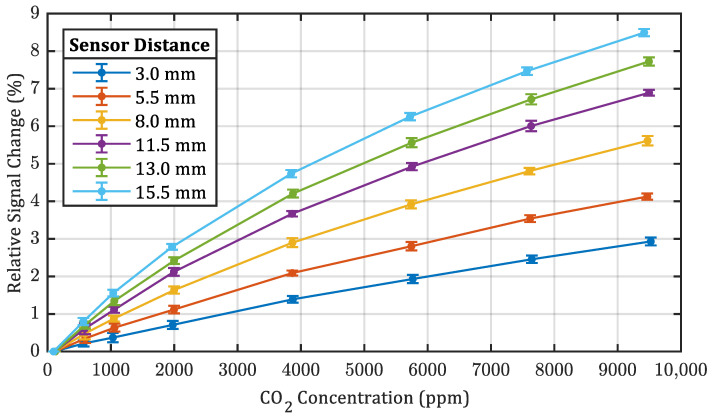
Signal response of six different absorption path distances ranging from 3.0 mm to 15.5 mm at different CO_2_ concentrations, relative to the the lowest reachable concentration. Error bars represent standard deviation.

We also characterized the same sensor prototype using six different absorption lengths. Figure 6 shows the relative signal response of all six sensor distances to seven different CO_2_ concentrations. The signal of the sample with the shortest absorption length dropped between 2.9% at a concentration of 9500 ppm to 0.2% at a concentration of 570 ppm. As expected from the Beer-Lambert law, the measurements with the longest absorption distances resulted in the highest signal response and those with the shortest absorption path in the smallest signal response. The signals of the other sensor lengths are laid in between those two configurations. As already done for the data of Figure 5, all values have been calculated from signal regions with stable CO_2_ concentrations and with a moving mean filter over 12 measurement periods applied. Although longer absorption paths provide higher sensitivity, they also result in larger setup sizes. For this reason, this study focused only on variants with an absorption path smaller or equal to 15.5 mm.

**Figure A3:**
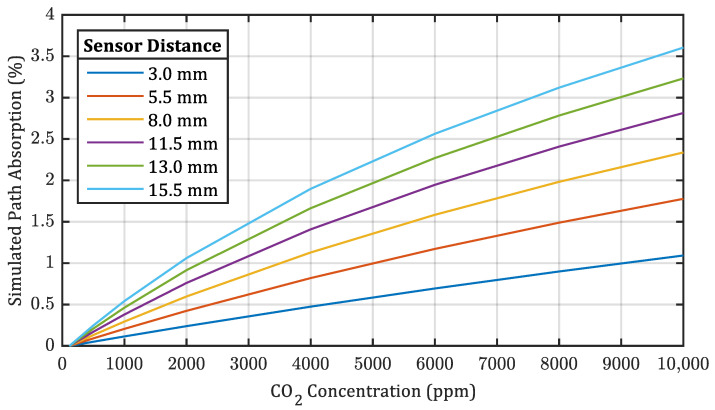
Additional absorption, simulated with HITRAN, for applied CO_2_ concentrations in the sensor path, normalized to the absorption of each absorption path at the measurements’ lowest CO_2_ concentration. Only the direct distance was calculated. Reflections inside the reflector tube were neglected.

Figure 6 also indicates an increasing non-linearity in terms of the relative signal response with increasing sensor distance. We assume this is mostly based on the natural non-linearity of the Beer-Lambert law, if calculated with non-monochromatic light. With increasing absorption path length and CO_2_ concentration, an increasing number of absorption lines in the CO_2_ spectrum gets fully absorbed within the absorption path, leading to an amplification of the non-linear effect. A simulation with HITRAN for the given concentrations and sensor distances confirmed this theory [23,24]. However, the simulation did not take into account any reflections inside the aluminum tube, which multiply the effective path lengths. The results are shown in the Appendix A in Figure A3.

**Figure 7:**
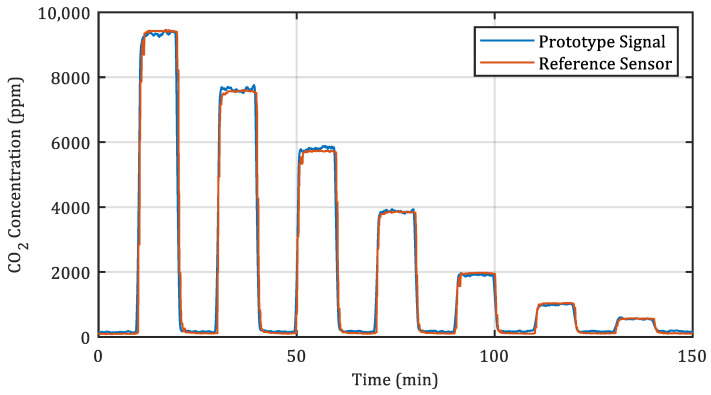
Calibrated sensor response absorption distance of d = 15.5 mm showing 10 min steps with decreasing CO_2_ concentration together with the output of an NDIR reference sensor (12-times moving mean applied).

**Figure A4:**
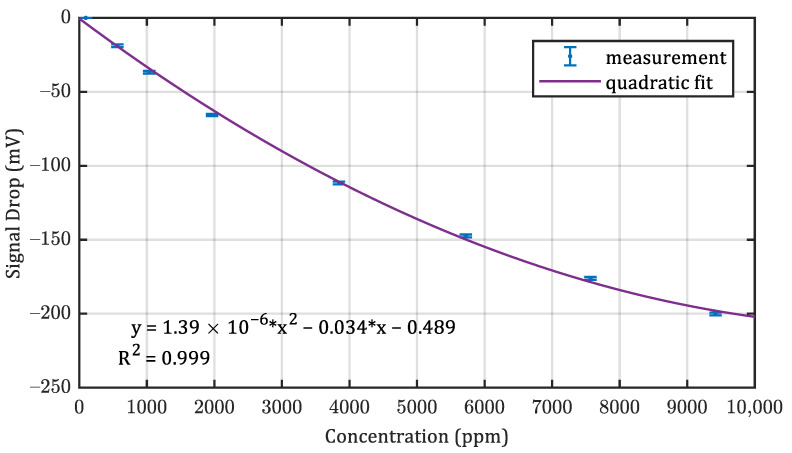
Absolute signal response and quadratic regression curve of d = 15.5 mm sample to applied CO_2_ concentrations. Error bars represent standard deviation.

In order to get a meaningful sensor output, which can be compared to other CO_2_ sensors, a basic quadratic regression curve was calculated from the measurements for the d = 15.5 mm sensor (Curve is depicted in the Appendix A, Figure A4). This calibration curve was then applied to the signal shown in Figure 5. The calibrated sensor response of this sample is depicted in Figure 7.

When applying the sensor’s sensitivity to a commonly occurring CO_2_ concentration of 1000 ppm ( −39.8 mV/1000 ppm), the standard deviation of the 15.5 mm sample was found to be equivalent to 18 ppm.

## 4. Conclusions

We presented a novel approach for manufacturing wafer-level photoacoustic gas sensors and provided the first proof-of-concept measurement results. Basic characterization of the built sensor modules at various absorption distances showed promising sensitivity and noise levels even at short absorption path lengths.

The photoacoustic transducer can be manufactured using only materials and process steps, which are already standard in the semiconductor industry and are therefore reasonably priced in production. Moreover, as in this approach, the acoustic detector can be manufactured, tested, and handled separately from the photoacoustic cell. As a result, the exposition to harsh environments or the need for non-standard manufacturing processes is limited to a minimum. Earlier studies with pressure or sound transducers directly encapsulated in the gas-filled cell, either needed to use non-standard low-temperature encapsulation processes in gas bias atmospheres individually manufactured samples or harsh process environments [25,26,27,28,29].

The concept could be adapted for a wide range of gasses with absorption bands that overlap with the high-transmissivity region of silicon, which was used for the IR entrance window. The only changes needed for such an adaption would be the substitution of the gas inside the absorbing cavity with another target gas and the exchange of the IR source or filter to form a matching pair of emitter and detector. Possible target gas candidates could include Methane or fluorine-based refrigerants [28,30]. Further experiments on the photoacoustic transducers themselves could benefit from a variation of the inner pressure, as this would allow a higher relative sensor response [30].

To our knowledge, this is the first successful approach that combines elements of photoacoustic and NDIR sensing enabling low-cost and well-performing IR detectors for CO_2_ sensors.

## Data Availability

Not applicable.
